# Hyperspectral Imaging Coupled with Random Frog and Calibration Models for Assessment of Total Soluble Solids in Mulberries

**DOI:** 10.1155/2015/343782

**Published:** 2015-09-14

**Authors:** Yan-Ru Zhao, Ke-Qiang Yu, Yong He

**Affiliations:** College of Biosystems Engineering and Food Science, Zhejiang University, 866 Yuhangtang Road, Hangzhou 310058, China

## Abstract

Chemometrics methods coupled with hyperspectral imaging technology in visible and near infrared (Vis/NIR) region (380–1030 nm) were introduced to assess total soluble solids (TSS) in mulberries. Hyperspectral images of 310 mulberries were acquired by hyperspectral reflectance imaging system (512 bands) and their corresponding TSS contents were measured by a Brix meter. Random frog (RF) method was used to select important wavelengths from the full wavelengths. TSS values in mulberry fruits were predicted by partial least squares regression (PLSR) and least-square support vector machine (LS-SVM) models based on full wavelengths and the selected important wavelengths. The optimal PLSR model with 23 important wavelengths was employed to visualise the spatial distribution of TSS in tested samples, and TSS concentrations in mulberries were revealed through the TSS spatial distribution. The results declared that hyperspectral imaging is promising for determining the spatial distribution of TSS content in mulberry fruits, which provides a reference for detecting the internal quality of fruits.

## 1. Introduction

Hyperspectral imaging, as a tool for spectrochemical analysis, integrates the advantage of conventional imaging and spectroscopic technique, which can obtain both spatial and spectral information from a tested object and has been widely used in detecting quality of fruit products [1]. Hyperspectral images, known as “hypercubes,” are made up of hundreds of contiguous wavebands for each spatial position of a target. “Hypercubes” are three-dimensional (*x* × *y* × *λ*) blocks of data, comprising two spatial dimensions (*x* and *y* direction) and one wavelength dimension (*λ*). Hyperspectral images often contain mass wavebands, which can result in modelling complicated. In addition, the existence of multicollinearity problem could reduce the accuracy of the calibration models. Feature transformation and variables selection are being able to reduce hyperspectral data dimension. Feature transformation is a process that creates a new set of features [2] and this method has been applied to extract feature in hyperspectral data. Zou et al. [3] used principal component analysis (PCA) and independent component analysis (ICA) to reduce the spectral dimension of the hyperspectral reflectance images of cucumber leaves and combined them with linear regression model to estimate chlorophyll concentration based on the extracted PCs and ICs. Yu et al. [4] implemented minimum noise fraction (MNF) rotation on important wavebands to extract the defective feature of hyperspectral images of loquat fruits and finally obtained that the identification accuracy was 92.3%. However, those methods only were used to eliminate useless information in view of spectra and neglect the relationship between spectral values and chemical concentrations. Feature transformation aims at preserving the topological structure of the data whereas the variables selection aims at enhancing the predictive power [2]. Recently, many studies proved that the selection of important variables can predigest calibration modelling and improve the results in terms of accuracy and robustness [5]. Moreover, selection of important wavelengths instead of full spectra of hyperspectral images has an advantage to generate chemical spatial distribution and provide a reference for developing portable multispectral imager [6]. Therefore, it is meaningful to extract important variables to establish robust calibration model.

Random frog (RF) methodology is a novel and efficient technique for variable selection, which borrows the framework like reversible jump Markov Chain Monte Carlo (RJMCMC) [7, 8]. It executes a search in the model space through both fixed-dimensional and transdimensional moves between different models, and then a pseudo-MCMC chain is computed and used to calculate selection probability (SP) for each variable. Important variables can be selected in terms of the ranking of all variables based on SP. RF has been used to select important wavelengths from spectral data in many studies. Hu et al. [9] used a combination of RF selected reflectance and transmittance spectra from hyperspectral data to predict blueberry mechanical properties, and the results showed that prediction models based on RF had similar results with full spectral model. Li et al. [10] detected tea polyphenols (TP) of 14 cultivars of tea using infrared spectroscopy with important wavenumbers selected by interval partial least squares (iPLS) combined with RF; finally, a linear formula with 18 wavenumbers provided satisfactory results for predicting TP measurement. Yu et al. [11] employed RF and partial least squares regression (PLSR) to establish calibration model for predicting total nitrogen content of pepper plant on the basis of hyperspectral imaging in the region of 380–1030 nm.

The “hypercube” could provide visualization of biochemical constituents of a sample by calculating the chemical value of each pixel based on the spectral prediction model. There are many ways to develop calibration models, such as principal components regression (PCR), multiple linear regression (MLR), partial least squares regression (PLSR), backpropagation neutral network (BPNN), and least-square support vector regression (LS-SVR). The widespread uses of PLSR make it possible to process visualization of hyperspectral images. Jin et al. [12] applied RCs-PLSR model to shift the spectrum of each pixel into its MC value for visualizing the MC distribution map in peanut kernels. *K* value spatial distribution map in grass carp and silver carp fillets was generated by employing the successive projection algorithm- (SPA-) PLSR model of the hyperspectral images (400–1000 nm) [13]. Simplified MLR model was used to visualize the thiobarbituric acid (TBA) values distribution in fish fillets and obtained good results (of 0.8395 and RMSE_*P*_ of 0.1147 mg MDA/kg flesh) [14]. Some authors attempted to realize chemical concentration visualization of hyperspectral images using nonlinear calibration model. For example, BPNN model considerably improved the performance of prediction set (*R*_*p*_^2^ of 0.938 and 0.965, RPD of 4.590 and 9.335) for detecting lycopene and total phenolic content in intact tomatoes and the BPNN model made it possible to predict the bioactive compounds in each pixel of the hyperspectral images [15]. Yang et al. [16] used hyperspectral imaging technique to detect different browning levels of lychee pericarp fruits that were affected by moisture contents. A few studies reported that SPA-LS-SVM was successfully applied to generate various indexes (freshness [17], total viable counts (TVC) [18], and Warner-Bratzler shear force (WBSF) [19]) distribution of meat for detecting quality of meat products. Other methods also could be used to map component distribution based on hyperspectral images; Siripatrawan and Makino [20] detected fungal infection on brown rice grains at early stage by using unsupervised self-organizing map (SOM) and visualized data classification of different levels of fungal infection.

Mulberry (*Fructus Mori*) fruits with bumpy surface and good taste were popular in food processing. Consumers' perception and satisfaction regarding fruit quality is an important issue in marketing [21]. Total soluble solids (TSS), which include the carbohydrates, organic acids, proteins, fats, and minerals, can contribute to the quality of fruits. Consequently, there is a need in the main producing industries to determine TSS rapidly and nondestructively to assure that fruits meet a minimum level of acceptance. Spectroscopic techniques have been proved to be an effective approach to detect the internal quality of fresh fruits [21]. Unfortunately, the spectroscopic technology fails to provide the quality parameters spatial information, which is essential to detail the analysis of the products' features [22]. What is more, based on the scanning mode, spectroscopic technology generally detects the small areas of fruits' surface and the information of the whole sample might be lost in the testing process. Visualization of chemical components using hyperspectral imaging has received an increasing attention in food-processing industry [23].

The specific objectives of this study were to (1) investigate the potential of Vis-NIR hyperspectral imaging to detect TSS of the mulberry fruits, (2) select the important wavelengths using RF algorithm, (3) evaluate the performance of the different calibration models with RF-PLSR and RF-LS-SVM based on linear and RBF kernel function, (4) compare the TSS visualization results which were developed with PLSR and LS-SVM and select the optimal calibration model, and (5) predict TSS value of each pixel in tested samples on the basis of the optimal calibration model and generate TSS spatial distribution.

## 2. Materials and Methods

### 2.1. Fruit Samples

Mulberries from local orchards (Hangzhou, Zhejiang, China) were selected for the research. The fruits were harvested randomly, ensuring a wide range of TSS. Prior to measurement, all 310 fruits were mature with the absence of any green area. Each single fruit constituted a sample, and as a result, 310 samples were collected and stored in the refrigerator at a constant temperature at 3°C in the laboratory before the hyperspectral images were acquired. Samples were removed from the refrigerator and placed under room condition (~20°C) for more than 2 hours.

### 2.2. Hyperspectral Image Acquisition and Calibration

**Figure 1:**
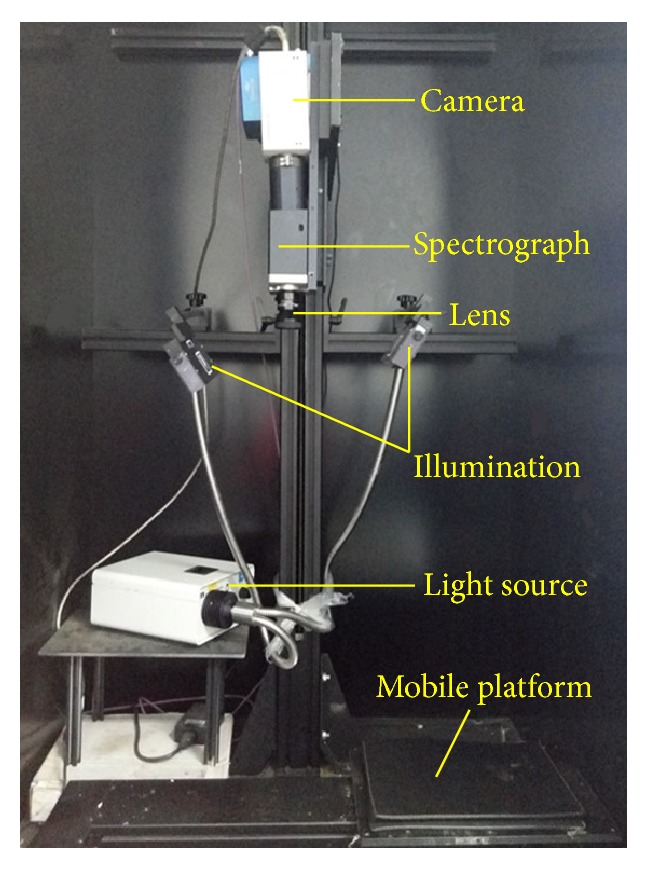
Schematic diagram of the main components of the hyperspectral imaging.

Mulberry samples were scanned by a push-broom hyperspectral imaging apparatus with reflectance mode as shown in Figure 1. The hyperspectral imaging system mainly consisted of an imaging spectrograph (ImSpectorV10E, Spectral Imaging Ltd., Finland) covering the spectral range of 380–1,030 nm; a CCD camera (C8484-05, Hamamatsu, Japan) coupled with a zoom lens (OLES23, Specim, Spectral Imaging Ltd., Oulu, Finland); an assembled illumination source coupled with two 150-W quartz tungsten halogen lamps (Fiber-Lite DC950 Illuminator, Dolan Jenner Industries Inc., USA); a mobile platform operated by a stepper motor (IRCP0076, Isuzu Optics Corp., Taiwan); and a computer with the spectral imaging system V10E software (Isuzu Optics Corp., Taiwan), which was used to set and adjust the parameters of the device, including exposure time, motor speed, imaging acquisition, wavelength range, and image correction. The spectral resolution is 2.8 nm; the resolution of CCD camera is 672 × 512 (spatial × spectral) pixels. Some parameters of apparatus for acquiring hyperspectral images should be set and adjusted before acquiring hyperspectral images of mulberries. In this work, the moving speed of mobile platform is 1.6 mm/s, exposure time of the CCD camera is 0.008 s, and the distance from the lens to samples is 295 mm. The whole system (except the computer) was assembled in a dark chamber to minimize the effects of ambient light during the sample scanning [4, 24].

Due to the existence of dark current in CCD camera and the uneven intensity of the light source in different bands, several bands with weaker light intensity contained the biggest noises [24]. Here, raw hyperspectral images were calibrated using the white and dark reference based on (1) for weakening the effect of dark current in the CCD camera and the uneven intensity of light in different bands:

(1) $$\begin{array}{c} I=\frac{I_{raw}-I_{dark}}{I_{white}-I_{dark}}, \end{array}$$

where *I* is the calibrated hyperspectral images, *I*_raw_ is the raw hyperspectral images, *I*_white_ is the white reference images (~99% reflectance), and *I*_dark_ is the dark reference images (~0% reflectance).

### 2.3. Image Processing

**Figure 2:**
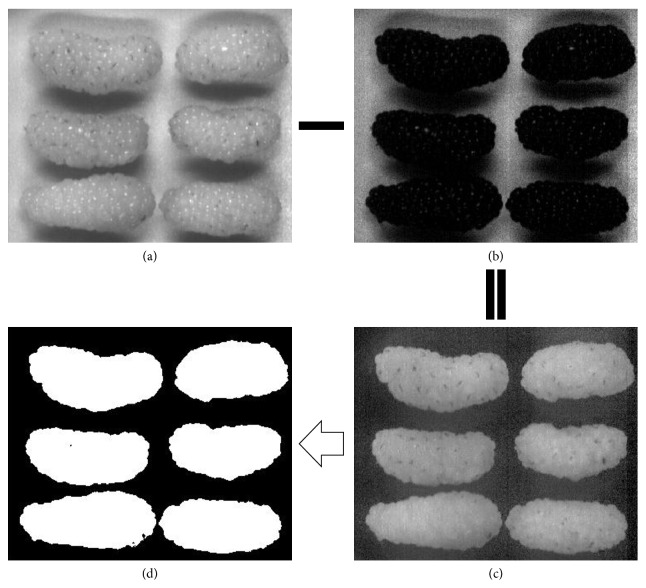
Details of acquiring mask (a) gray image at 893 nm; (b) gray image at 569** **nm; (c) imsubtract result of (a) and (b); (d) mask image.

Hyperspectral data were extracted from the calibrated hyperspectral images using ENVI software (version 4.6, ITT Visual Information Solutions, Boulder, USA). Before acquiring accurate spectra of the samples, background information should be removed in batches and this process has shorter processing time than manually extracted ROI of the individual sample. “Imsubtract” and “Threshold” algorithm were employed to create the mask. The details are displayed in Figure 2. The results subtracted from waveband at 893 nm (a) and 569 nm (b) are shown in Figure 2(c), in which it was found that there were big differences on gray value between tested samples and background images. Then threshold of 0.4 was used to remove the background and good masks (d) were obtained.

By implementing the masks in the original hyperspectral images, the background information was removed. The separated region was identified as the region of interest (ROI) of the sample. The average spectrum of spectra of all pixels in a mulberry was considered as the spectrum of a sample and 310 spectrums were collected. To avoid the low signal-noise ratio and diminish the problem of high dimensionality of feature spaces, the wavebands of 420–1,000 nm were considered in the analysis [11]. Ultimately, a spectral data matrix of 310 × 460 (samples × wavebands) was obtained for further analysis.

### 2.4. Detection of TSS

Total soluble solids (TSS, °Brix%) in mulberry samples were measured using traditional destructive tests. After acquiring the hyperspectral data, each fruit unit was juiced and TSS were measured by using WAY-2S Digital Refractometer (Shanghai Precision & Scientific Instrument Co., Ltd., Shanghai, China). The instrument range covered from 0 to 95% with temperature correction and refractive index accuracy is ±0.0002 [25]. All measurements were averaged over the data from three replicates in a room at 20°C.

### 2.5. Chemometrics Methods

#### 2.5.1. Random Frog Algorithm

**Figure 3:**
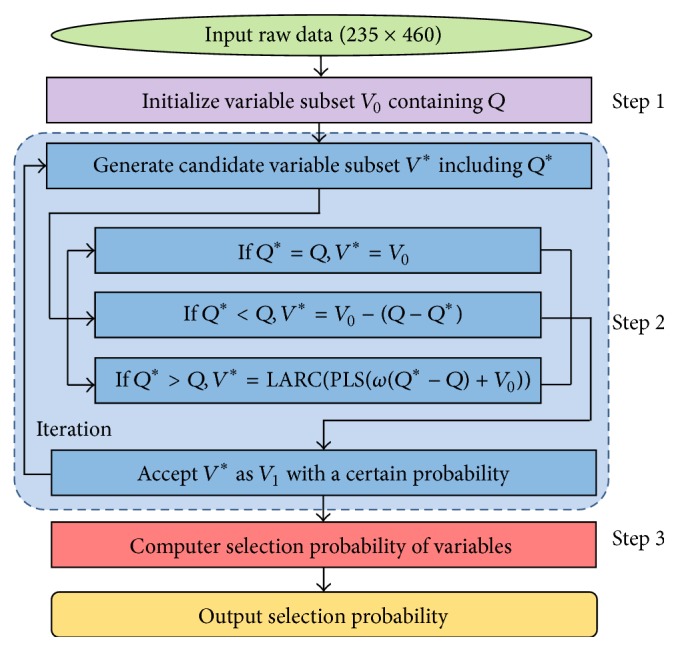
The flowchart of the random frog algorithm. Step  1: a variable subset *V*_0_ containing *Q* variables is initialized randomly; Step  2: candidate variable subset *V* ^*∗*^ including *Q* ^*∗*^ variables is generated according to the normal distribution norm (*Q*, *θQ*); Step  3: compute a selection probability of each variable.

The key steps of RF are illustrated in Figure 3 and the detailed algorithm of RF was described in literatures [7, 8]. Before running RF algorithm, five parameters (*T*, *Q*, *θ*, *ω*, and *η*) should be assigned to proper values. *T* was the number of iterations and needed to be sufficiently large to achieve convergence (*T* = 10,000); *Q* was the number of variables in the initialized variables set (*Q* = 50); *θ* controlled variance of a normal distribution (*θ* = 0.3); *ω* was a coefficient explained in Step  2 (*ω* = 3); *η* represented the upper bound of the probability (*η* = 0.1) [7].

#### 2.5.2. Partial Least Squares Regression

Partial least squares regression (PLSR) is a multivariate data analysis technique which generalizes and combines features from principal component analysis (PCA) and multiple linear regression (MLR) [26]. PLSR has been successfully used in developing multivariate calibration models, as it uses the concentration information (**Y**) in determining how regression factors are computed from the spectral data matrix (**X**), thereby reducing the impact of irrelevant *X*-variations in the calibration model. The important feature of PLSR is based on latent variables (LVs) [27].

There were three steps to develop PLSR; the first step is to decompose the matrix and the model is

(2) $$\begin{array}{c} X=TP+E,\\ Y=UQ+F, \end{array}$$

where **T** and **U** are the score matrices of **X** matrix and **Y** matrix, **P** and **Q** are the loading matrices of **X** matrix and **Y** matrix, and **E** and **F** are the errors which come from the process of PLS.

The second step is to process **T** and **U** by linear regression. It must build the following linear correlation:

(3) $$\begin{array}{c} U=BT+E, \end{array}$$

where **B** = (**T** ^*T*^ **T**)^−1^ **T** ^**T**^ **Y**.

Finally, unknown **Y** is predicted in the following PLSR model:

(4) $$\begin{array}{c} Y_{P}=T_{P}BQ. \end{array}$$

#### 2.5.3. Least-Square Support Vector Machine

LS-SVM is an alternate formulation of SVM regression proposed by Suykens et al. [28]. The main advantage is that it is computationally more efficient than the standard SVM method. The details of LS-SVM algorithm were introduced as follows [5].

Optimization problem of LS-SVM is formulated:

(5) $$\begin{array}{c} \min J\left(w,e\right)=\frac{1}{2}w^{T}w+\frac{1}{2}\gamma \overset{N}{\underset{k=1}{\sum }}e_{k}^{2} \end{array}$$

subject to the constraints

(6) $$\begin{array}{c} y_{k}=w^{T}\varphi \left(x_{k}\right)+b+e_{k},\;k=1,\ldots ,N, \end{array}$$

where *γ* is the regularization parameter which balances the model's complexity and the training errors; *e*_*k*_ is the random error; *x*_*k*_ and *y*_*k*_ are input and output variables; *k* is sample number.

And then, Lagrange function is applied to solve the optimization problem

(7) Lw,b,e,α=Jw,e−∑k=1NαkwTφxk+b+ek−yk,

where *α*_*k*_ ∈ *R* is Lagrange multipliers. The solution of the above equation can be obtained by partially differentiating with respect to each variable:

(8) $$\begin{array}{c} \frac{\partial L}{\partial w}=0⟶w=\overset{N}{\underset{k=1}{\sum }}\alpha_{k}\varphi \left(x_{k}\right),\\ \frac{\partial L}{\partial b}=0⟶\overset{N}{\underset{k=1}{\sum }}\alpha_{k}=0,\\ \frac{\partial L}{\partial e_{k}}=0⟶\alpha_{k}=\gamma e_{k},\;k=1,\ldots ,N,\\ \frac{\partial L}{\partial \alpha_{k}}=0⟶w^{T}\varphi \left(x_{k}\right)+b+e_{k}-y_{k}=0,\;k=1,\ldots ,N. \end{array}$$

When the variables *w* and *e* are removed, the equation can be rewritten as a linear function group

(9) $$\begin{array}{c} \left[\begin{array}{cc} 0 & {\vec{1}}^{T}\\ \vec{1} & \Omega +\gamma^{-1}I \end{array}\right]\left[\begin{array}{c} b\\ a \end{array}\right]=\left[\begin{array}{c} 0\\ y \end{array}\right] \end{array}$$

with

(10) $$\begin{array}{c} y=\left[y_{1},\ldots ,y_{N}\right],\\ \vec{1}=\left[1,\ldots ,1\right],\\ \alpha =\left[\alpha_{1},\ldots ,\alpha_{N}\right],\\ \Omega =\left\{\Omega_{kl}\;|\;k,l=1,\ldots ,N\right\},\\ \;\Omega_{kl}=\varphi {\left(x_{k}\right)}^{T}\left(x_{l}\right)=K\left(x_{k},x_{l}\right),\;\;k,l=1,\ldots ,N, \end{array}$$

where *K*  (*x*_*i*_, *x*_*j*_) is defined as the kernel function and must satisfy Mercer's condition.

Kernel function can map sample in original space to high-dimensional feature space to solve the linear inseparable problem [29]. There are several typical examples of kernel function such as linear kernel, polynomial kernel, RBF, and sigmoid kernel. Each kernel has some parameters, while RBF kernel function is strongly recommended and widely used for its performance and complexity [30]. Linear kernels usually compute fast. LS-SVM with RBF kernel and linear function were selected in our work to compare the predictive performance with PLSR.

The LS-SVM regression model can be obtained as

(11) $$\begin{array}{c} y\left(x\right)=\overset{N}{\underset{k=1}{\sum }}\alpha_{k}K\left(x,x_{k}\right)+b. \end{array}$$

Grid-research and leave-one-out cross-validation were used to find out the optimal *γ* (gam) value and *σ* ^2^ (sig2), which is the bandwidth in the case of the RBF kernel. *γ* is the regularization parameter, determining the trade-off between structural risk minimization principle and empirical risk minimization, and is important to improve the generalization performance of LS-SVM models, while *σ* ^2^ controls the value of function regression error and influences the number of initial eigenvalues [5] and *σ* ^2^ is absent in linear kernel function. In this case, we use leave-one-out CV to determine the tuning parameters.

### 2.6. Evaluation of Model Performance

The performances of models were evaluated using correlation coefficient (*R*) and root mean square error (RMSE) in calibration set (*R*_*C*_, RMSE_*C*_), cross-validation set (*R*_CV_, RMSE_CV_), and prediction set (*R*_*P*_, RMSE_*P*_) [31]. Generally, an optimal model should offer high *R* values and low RMSE values; small difference existed between calibration and prediction set.

### 2.7. Chemical Imaging Process

**Figure 4:**
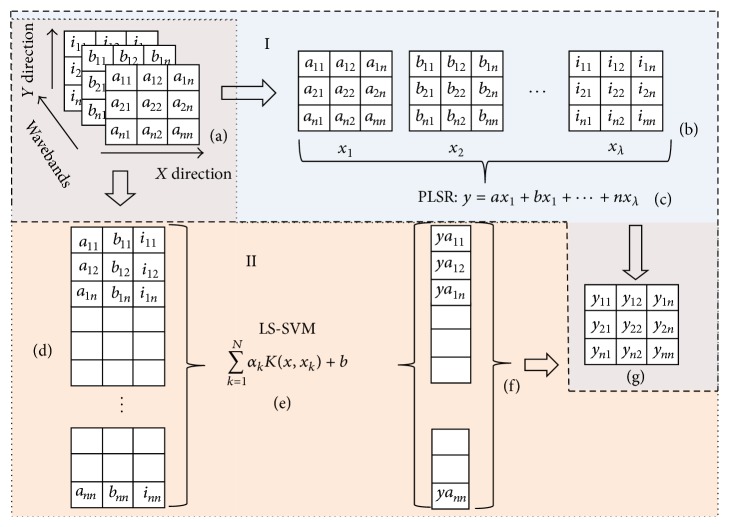
Visualization flowchart based on PLSR and LS-SVM.

The optimized model was employed to predict TSS of mulberries. As shown in Figure 4, there were two paths to develop the visualization map in this study. In method (I), the hyperspectral image of a sample is a 3D data cube (*x* × *y* × *λ*) (a), and there are *n* pixels in *x* and *y* direction, respectively. First, unfold the three-dimensional data matrix into a two-dimensional (*n* × *λn*) matrix and the data matrix with *n* × *n* pixels being defined as variable *X*_*i*_ (b), and PLSR model (c) was applied to predict the chemical value of each pixel, forming a prediction image (g). In method (II), the pixels at the same position at important wavebands were extracted and arranged in a row, and all pixels were arranged at column; in all, *i* × *n* ^2^ data matrix (d) was formed. LS-SVM was used to calculate TSS values of pixels, 1 × *n* ^2^ data (e) was figured out, and then (e) was folded into a *n* × *n* matrix (f), namely, the image of the tested sample with predictive TSS value. Pseudo-colour images were created with different colours representing different levels of TSS that were predicted by the optimal simplified model [32]. Useful information about TSS distribution in mulberries was observed by checking chemical images.

There are many kinds of noise in hyperspectral images, such as electrical noise from CCD detector; the noise was caused by transmitting procedure and others. The presence of the noise seriously affects the feature extraction and recognition accuracy of the tested objects. It is necessary to process image denoising before conducting further analysis. Median filtering, in which gray value of every pixel is set to be the average gray value in a certain neighbourhood window, is a nonlinear filtering technique that has been successfully applied to many signal and image processing tasks [33]. Median filtering (5 × 5) was applied to denoise hyperspectral images in this study.

### 2.8. Software

Random frog (version 2.0) toolbox, LS-SVM lab v1.8 toolbox, median filtering toolbox, and the visualization program were finished with MATLAB R2009a (version 7.8) software. PLSR models were established using “The Unscrambler X10.1” (CAMO PROCESS AS, Oslo, Norway). Origin Pro 8.0 SR0 (Origin Lab Corporation, Northampton, MA, USA) software was employed to design the curve figures. All the processes were run on a PC (CPU: G2020 @ 2.90 GHz, RAM 4.00 GB) operating on Windows 7.

## 3. Results and Discussion

### 3.1. Analysis of the Measured TSS

In order to establish the calibration models of the TSS in mulberries, all mulberry samples were divided into a calibration set and prediction set according the ratio about 3 : 1 (235 : 75) by SPXY method, in which sample set partitioning is based on joint *x*-*y* distances [34].  Table 1 summarized the measured TSS of all samples, the maximum and minimum value of calibration set were 10.99°Brix and 3.21°Brix, and the range of prediction set was 9.86–3.86°Brix. Usually, calibration set with a wide range of the chemical component could establish a robust model.

### 3.2. Feature of Spectra

**Figure 5:**
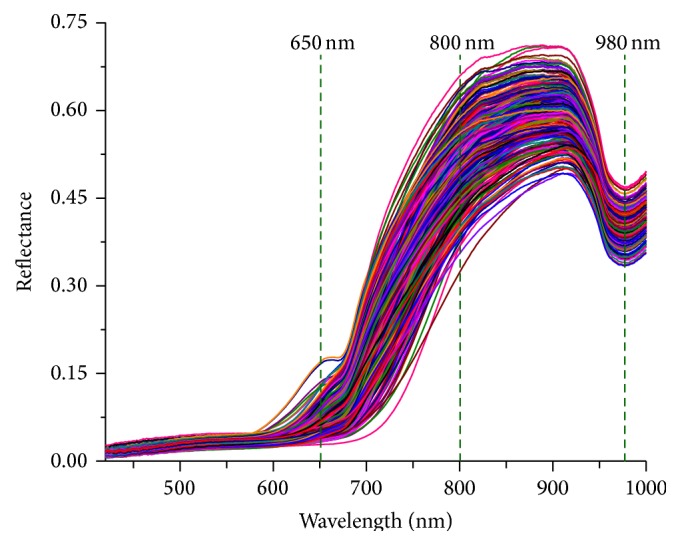
Spectral curves of all mulberries covering the range of 420–1,000 nm.

Spectra of all mulberries covering the range of 420–1,000 nm are displayed in Figure 5. Lower reflectance (<10%) in the visible region of 420–650 nm was attributed to the relatively homogeneous and dark purple or black colour of mature fruits and mainly caused by anthocyanin and chlorophyll [35, 36]. Reflectance for mulberry samples started to increase dramatically from 650 to 800 nm and reached a peak at 850 nm. An obvious valley located around 960–980 nm, which was attributed to the combination effect of -OH groups from carbohydrate and water [37]. This fact has also been reported in the case of Huang et al. [25]. Chemometrics methods were introduced to analyze the spectra and establish the relationship between spectra and measured TSS to determine the internal quality of mulberries in the future study.

### 3.3. Selection of the Important Wavelengths

**Figure 6:**
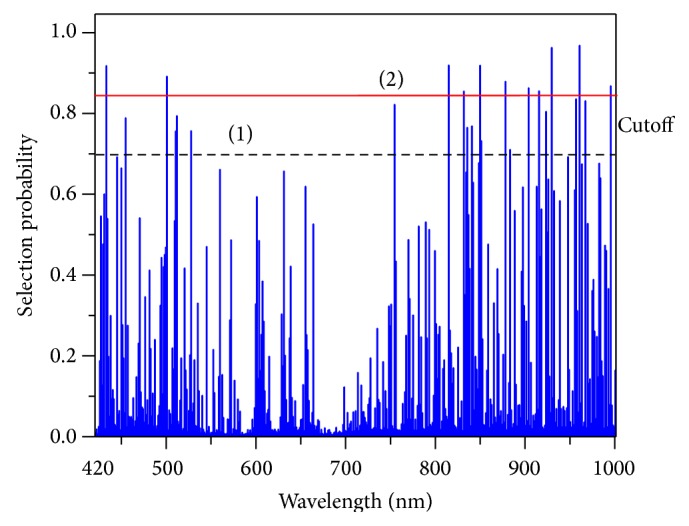
Selection probability of each wavelength.

RF was used to select important wavelengths from original data.  Figure 6 displays SPs of wavelengths, and a small number of wavelengths displayed had high SP (over 0.9); most of the wavelengths were with low SP, and these results showed that there existed a lot of useless information to make TSS content be predicted using hyperspectral imaging. If the cutoff of SP was 0.7 (1) and 0.85 (2), there were 23 (960, 929, 814, 849, 432, 500, 877, 995, 903, 915, 831, 956, 967, 954, 923, 511, 454, 840, 835, 527, 509, 850, and 883 nm) and 11 (960, 929, 814, 849, 432, 500, 877, 995, 903, 915, and 831 nm) important wavelengths selected, respectively. These wavebands were ranked by descending order of SPs. These wavelengths were mainly concentrated on two parts: 400–550 and 800–1,000 nm. The concentration of anthocyanin and chlorophyll caused low reference in the visible region and contributed to the maturity of mulberry fruits. Wavelengths at 814 nm, 975–989 nm, and 981 nm correspond to second and third overtones of –NH, 877 nm and 900–950 nm were assigned to –CH third overtone, and 960 nm was assigned to –OH second overtone. These bands are assigned to mono- and ploy-carbohydrates (fructose, glucose, and pectin) and water in mulberry [36].

In order to establish calibration models with fewer features, two important wavelengths sets (23 versus 11) were employed to establish calibration models and these results were compared to obtain optimal visualization map of TSS in mulberry.

### 3.4. Calibration Models

Multivariate analyses, developed with leave-one-out CV, were used to find accurate PLSR and LS-SVM models for the prediction of TSS. The predictive models of TSS in mulberry fruits were built using the two kinds of the selected wavelengths (23 versus 11) and the results of these models are enumerated in Table 2.

Overall, LS-SVM regression models had better performance for predicting TSS than PLSR models because LS-SVM is a nonlinear regression model and it could transform the original data into a high dimension space to make linear solution [38]. LS-SVM was capable of solving the nonlinear problem of the calibration models. LS-SVM with RBF kernel function based on 23 wavelengths with *R*_*P*_ of 0.956 and RMSE_*P*_ of 0.430 could provide the most effective TSS estimation compared to other models, while LS-SVM models with linear function had similar results with RF-PLSR models (except with full wavelengths). This consequence caused by the RBF kernel function of LS-SVM has an advantage in conducting samples in multidimensional space. In addition, linear kernel function in LS-SVM was considered as a special form of RBF kernel function [39].

LS-SVM with fewer wavelengths was able to provide more accurate results. Model (9) with 11 important wavelengths had an approving expression with *R*_*P*_ of 0.925 and RMSE_*P*_ of 0.557, and these results were equal to the performance of the full-PLSR model (*R*_*P*_ of 0.959 and RMSE_*P*_ of 0.411). When the number of important wavelengths reduced to almost a half (11 versus 23), model (9) only had a little reduction of 3.24% (*R*_*p*_) and an increase of 29.5% (RMSE_*P*_) compared to model (6). Hence, model (9) is appropriate to predict TSS in mulberry fruits.

Although LS-SVM had better performance, PLSR with 23 important wavelengths offered acceptable results. Model (4) provided a reliable result of *R*_*P*_ of 0.899 and RMSE_*P*_ of 0.675. Compared with full-PLSR model, *R*_*C*_, *R*_CV_, and *R*_*P*_ in model (4) showed a slight reduction of 3.88%, 2.52%, and 5.47%; RMSE_*C*_, RMSE_CV_, and RMSE_*P*_ provided a change of 0.213, 0.101, and 0.228, respectively. However, 95.0% of the variables (23 versus 460) were removed in RF-PLSR model. RF-PLSR model could provide a more effective prediction for TSS of mulberries. The accuracy of RF-PLSR model was higher than SPA-MLR model in the literature of [25] for predicting TSS of mulberries, because hyperspectral imaging could provide both spectra and image information about tested samples. In addition, spectra of whole fruit were averaged as the spectrum of the sample to avoid the loss of spectra. But beyond that, there were only two wavelengths in the SPA-MLR model which meant that useful spectral information might be over-removed. However, RF-PLSR model for predicting TSS of mulberries was not as good as Monte Carlo-uninformative variable elimination- (MC-UVE-) SPA (MC-UVE-SPA) model for predicting TSS of “Ya” pear [31]. This phenomenon could be explained that the mulberry fruit has a bumpy surface that has more influence on the spectra than does a smooth surface [25]. TSS prediction of mulberries based on RF-PLSR model was parallel to the prediction TSS of blueberries that were acquired by interval partial least square- (iPLS-) PLSR model [35].

### 3.5. Distribution Map of TSS in Mulberries

**Figure 7:**
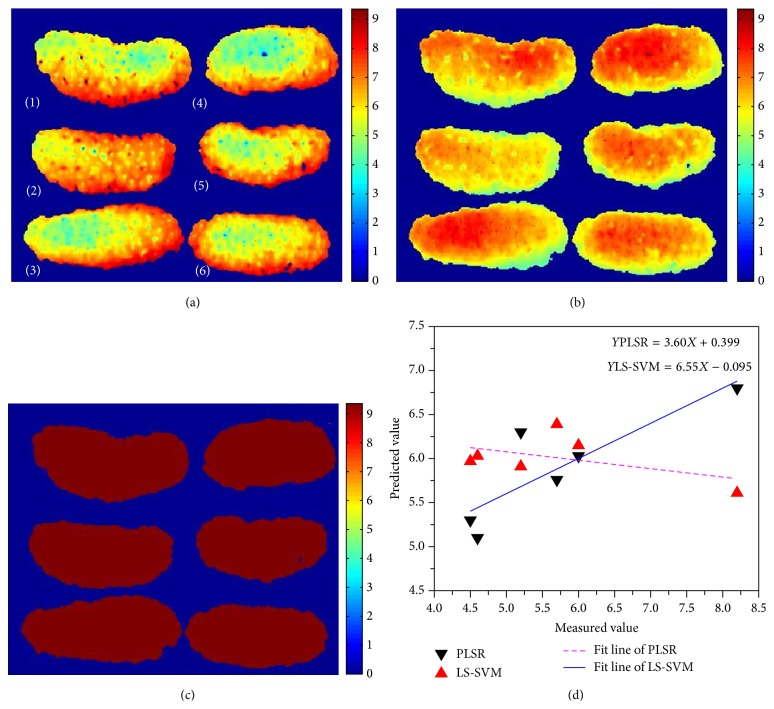
Compared TSS distribution results with PLSR and LS-SVM models: (a) distribution map using PLSR model; (b) visualization result based on LS-SVM model with linear kernel function; (c) TSS distribution using LS-SVM with RBF kernel function; (d) fitting effect between measured and predicted results of PLSR and LS-SVM with linear kernel function. Colour bar represents the TSS concentration.

In order to seek an optimal calibration model for realizing TSS visualization in mulberry fruits, models (4), (8), and (9) were employed to process visualization procedure. The comparative maps of six samples developed by three models are exhibited in Figure 7.

Six mulberry fruits were used to compare the reliability of TSS distribution maps, which were predicted by three models. PLSR (Figure 7(a)) and LS-SVM with linear kernel function (Figure 7(b)) could provide clearly TSS distribution, while LS-SVM with RBF kernel function (Figure 7(c)) failed to display TSS visualization of mulberry fruits. There are four possible reasons to explain this phenomenon: (1) the special modelling way of LS-SVM with RBF kernel function, which needed to transfer the raw data into high-dimensional space, might change the original data form; (2) there were two parameters (*γ*, *σ* ^2^) to control the predicted results of LS-SVM, which might add the complexity of LS-SVM; (3) using calibration model with 235 variables to predict a map (such a map of a mulberry was about 100 × 200 pixels) might produce problem of overfitting; (4) the mulberry fruit with bumpy appearance would bring the variation of spectral reflectance, which might affect the accuracy of models.

The linear kernel function is the special form of RBF kernel function, and LS-SVM with linear kernel function (LS-SVM-linear) (b) had better TSS distribution (b) than that of LS-SVM-RBF. However, in Figure 7(b), it was hard to distinguish the TSS level of mulberry and a mistake was made in sample (2). The measured TSS value of sample (2) was 8.2°Brix and it was the highest TSS value among these six samples, while in Figure 7(b), it had the lightest colour. PLSR had the best performance compared to LS-SVM for its simple linear combination. At the same time, the fitting effects of PLSR and LS-SVM with linear kernel function (LS-SVM-linear) were compared and expressed in Figure 7(d). The correlation coefficients between measured TSS values and predicted TSS values in PLSR and LS-SVM with linear kernel function (LS-SVM-linear) were 0.857 and −0.500, respectively. Although LS-SVM could offer satisfactory results in calibration model, PLSR with simple algorithm was feasible to map TSS distribution in mulberry fruits. When the simplified model was finally established, it was subsequently employed to predict TSS in each pixel of the image resulting in new pseudo-colour images and this process was called “prediction map” [32]. As the last step of analyzing hyperspectral image, RF-PLSR model was used to predict TSS of each pixel and transferred its hyperspectral image to the TSS distribution map.

The multilinear function for the TSS prediction of the mulberries was obtained:

(12) YTSS=16.207−0.191λ1−0.818λ2−1.717λ3−1.481λ4+0.007λ5+0.037λ6−1.291λ7−0.193λ8−1.112λ9−1.017λ10−1.606λ11−0.230λ12−0.168λ13−0.132λ14−0.924λ15+0.042λ16+0.023λ17−1.541λ18−1.574λ19+0.047λ20+0.040λ21−1.474λ22−1.252λ23,

where *λ*_*i*_ is the spectral reflectance value at the wavelength of *i* nm (*i* was 960, 929, 814, 849, 432, 500, 877, 995, 903, 915, 831, 956, 967, 954, 923, 511, 454, 840, 835, 527, 509, 850, and 883, resp.) and *Y*_TSS_ is the predictive TSS values of the mulberry fruits.

**Figure 8:**
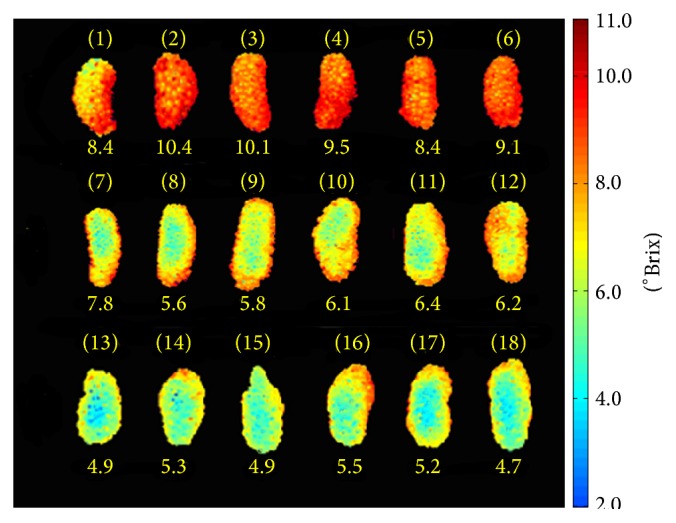
TSS visualization of mulberry fruits.

New hyperspectral data with only 23 wavelengths could speed up the visualization process and make it easier to establish a multispectral imaging system. The function (12) was employed to predict the TSS of each pixel within the mulberry fruits hyperspectral images. The spatial distribution maps of samples' TSS are generated in Figure 8.

The predicted TSS concentration of each pixel was mapped with a linear colour scale using different colours from red to blue to represent different TSS concentrations from high to low. Mulberries with higher TSS values have more pixels shown in red, such as in Figure 8 (1–6) samples. The average TSS value of these six samples was 9.3°Brix. The more the pixels coloured in green and blue, the lower the TSS values. There were unsatisfying results: the blue pixels in samples (13) and (14) were saturated points and were not considered in the analysis [40].

Different values in the maps of TSS distribution were in quantitative proportion to the spectrum of the corresponding pixels. However, these distribution maps are difficult to be inspected by naked eyes [41]. The ability to provide spatial information makes hyperspectral imaging available to focus on detecting both external and internal quality of fruits.

## 4. Conclusions

To sum up, the successful mapping of TSS distribution in mulberries suggested that the application of hyperspectral imaging to realize the visualization of mulberry fruits' internal quality is feasible and promising. The PLSR and LS-SVM model based on 23 and 11 wavelengths had a good performance to predict TSS of mulberries, which indicated that RF algorithm was effective in reducing three-dimensional data. PLSR-RF based on 23 important wavelengths provided the optimal visualization results. It could be revealed that PLSR was feasible to map chemical component concentration (TSS) distribution of mulberry fruits. This research provided a theoretical basis for developing the instrument for measuring the internal quality of fruits and made it possible to sort mulberries based on TSS spatial distribution.

## Figures and Tables

**Table 1:** Summary of the measured TSS in samples of calibration and prediction set (°Brix).

Group	Range	Mean	SD^a^
Calibration	10.99–3.21	6.86	1.56
Prediction	9.86–3.86	6.35	1.45
Total	10.99–3.21	6.75	1.55

^a^Standard deviation of each group.

**Table 2:** Results of TSS predictive models of mulberries.

Number	Models	No.^c^	LVs^d^	Calibration		Cross-validation		Prediction		*γ*(10^3^)	*σ* ^2^(10^3^)
				*R*_*C*_	RMSE_*C*_	*R*_CV_	RMSE_CV_	*R*_*P*_	RMSE_*P*_		
(1)	Full-PLSR	460	21	0.984	0.279	0.958	0.445	0.959	0.411		
(2)	Full-LS-SVM-RBF	460		0.999	<0.1	0.999	<0.1	0.999	<0.1	4.43 × 10^5^	
(3)	Full-LS-SVM-linear	460		0.999	<0.1	0.999	<0.1	0.999	<0.1	1.11 × 10^8^	8.99 × 10^2^
(4)	RF-PLSR	23	13	0.942	0.522	0.928	0.579	0.899	0.675		
(5)	RF-LS-SVM-linear	23		0.944	0.515	0.931	0.571	0.886	0.714	4.93	
(6)	RF-LS-SVM-RBF	23		0.999	0.061	0.958	0.453	0.956	0.430	9.56	4.29
(7)	RF-PLSR	11	7	0.811	0.912	0.792	0.951	0.834	0.800		
(8)	RF-LS-SVM-linear	11		0.818	0.899	0.796	0.945	0.843	0.781	0.36	
(9)	RF-LS-SVM-RBF	11		0.984	0.283	0.912	0.647	0.925	0.557	1.25 × 10^8^	1.80

^c^Numbers of the wavelengths used for analysis.

^d^Latent variables of the PLSR model.
